# IPF-related new macrophage subpopulations and diagnostic biomarker identification - combine machine learning with single-cell analysis

**DOI:** 10.1186/s12931-024-02845-8

**Published:** 2024-06-13

**Authors:** Hao Zhang, Yuwei Yang, Yan Cao, Jingzhi Guan

**Affiliations:** 1 Department of Oncology, The Eighth Medical Center, Chinese PLA (People’s Liberation Army) General Hospital, Beijing, 100091 China; 2 Department of Oncology, The Fifth Medical Center, Chinese PLA (People’s Liberation Army) General Hospital, Beijing, 100071 China; 3 College of Pulmonary & Critical Care Medicine, Chinese PLA General Hospital, Beijing, 100091 China; 4 Beijing Key Laboratory of OTIR, Beijing, 100091 China

**Keywords:** Idiopathic pulmonary fibrosis, Lung macrophages, Machine learning, Prediction models, Single-cell RNA sequencing, hdWGCNA

## Abstract

Idiopathic pulmonary fibrosis (IPF) is a chronic disease of unknown etiology that lacks a specific treatment. In IPF, macrophages play a key regulatory role as a major component of the lung immune system, especially during inflammation and fibrosis. However, our understanding of the cellular heterogeneity and molecular characterization of macrophages in IPF, as well as their relevance in the clinical setting, is relatively limited. In this study, we analyzed in-depth single-cell transcriptome sequencing (scRNA-seq) data from lung tissues of IPF patients, identified macrophage subpopulations in IPF, and probed their molecular characteristics and biological functions. hdWGCNA identified co-expressed gene modules of a subpopulation of IPF-associated macrophages (IPF-MΦ), and probed the IPF-MΦ by a machine-learning approach. hdWGCNA identified a subpopulation of IPF-associated macrophage subpopulations and probed the IPF-MΦ signature gene (IRMG) for its prognostic value, and a prediction model was developed on this basis. In addition, IPF-MΦ was obtained after recluster analysis of macrophages in IPF lung tissues. Coexpressed gene modules of IPF-MΦ were identified by hdWGCNA. Then, a machine learning approach was utilized to reveal the characteristic genes of IPF-MΦ, and a prediction model was built on this basis. In addition, we discovered a type of macrophage unique to IPF lung tissue named ATP5-MΦ. Its characteristic gene encodes a subunit of the mitochondrial ATP synthase complex, which is closely related to oxidative phosphorylation and proton transmembrane transport, suggesting that ATP5-MΦ may have higher ATP synthesis capacity in IPF lung tissue. This study provides new insights into the pathogenesis of IPF and provides a basis for evaluating disease prognosis and predictive medicine in IPF patients.

**Graphical Abstract:**

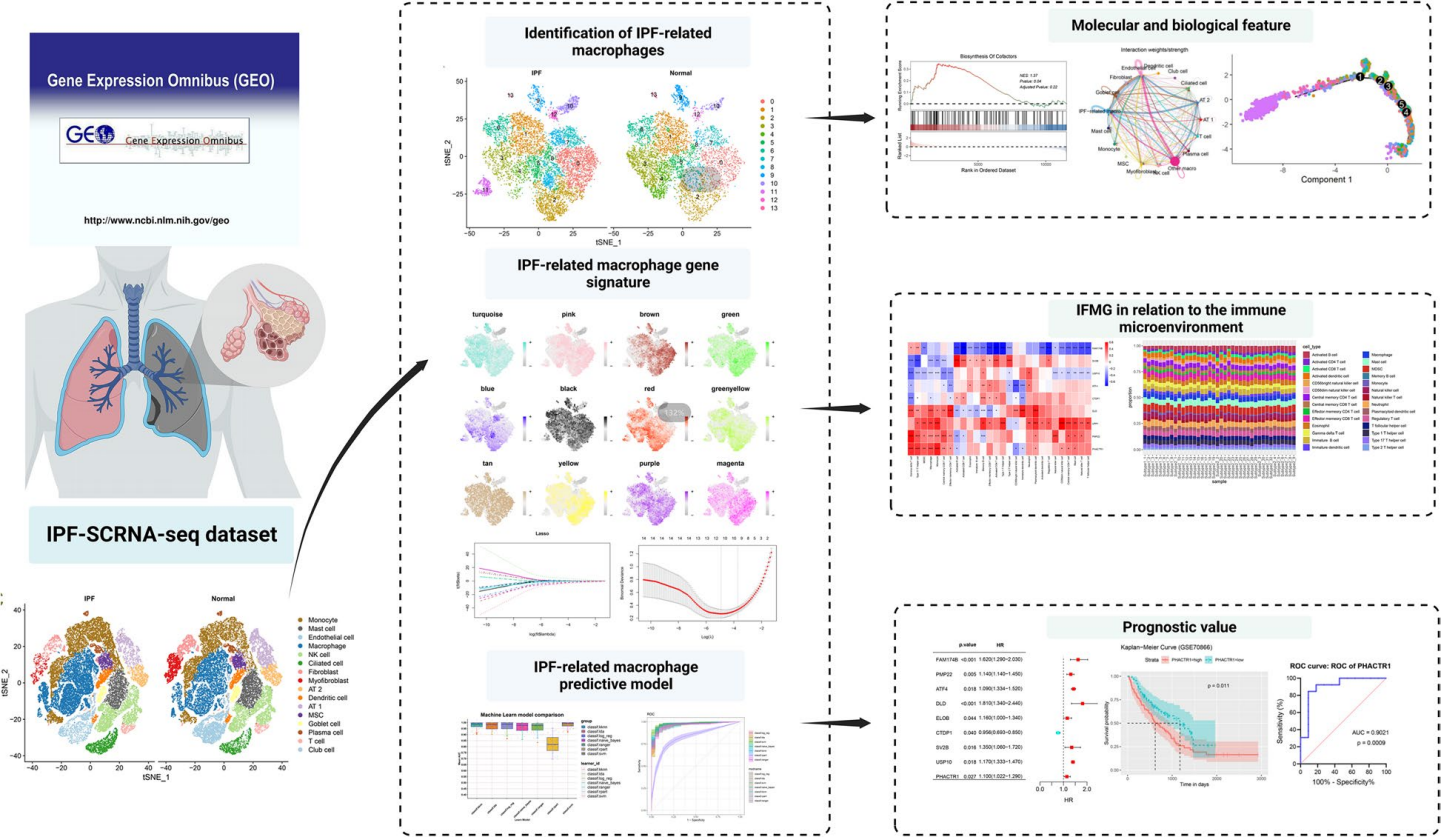

**Supplementary Information:**

The online version contains supplementary material available at 10.1186/s12931-024-02845-8.

## Introduction

Idiopathic pulmonary fibrosis (IPF) is a rare, progressive, and fatal lung disease [1]. The incidence of IPF, although low globally [2], is notable for its high mortality rate, with an average life expectancy of only 3–5 years [3]. Currently, patients with IPF require supportive therapy such as oxygen therapy to alleviate hypoxemia and to reduce shortness of breath. There are a limited number of FDA-approved antifibrotic medications for the treatment of IPF, including only pirfenidone and nintedanib. Both can slow the decline of lung function in IPF patients, and nintedanib in particular significantly reduces the risk of acute exacerbations [4, 5]. After discontinuation of these two drugs, patients’ lung function deteriorates [5]. However, pirfenidone and nintedanib do not reverse the progression of the disease, and therefore, lung transplantation has become the only treatment available [6–8]. Given this background, the search for more effective therapeutic strategies has become an urgent task in the field of IPF.

IPF is not a single pathophysiologic process but is the result of a combination of mechanisms [9]. A combination of genetic, environmental, and aging factors leads to epithelial cell damage and aberrant activation. Abnormally reprogrammed epithelial cells activate fibroblasts to differentiate into myofibroblasts, and persistently activated fibroblasts and myofibroblasts secrete large amounts of extracellular mesenchyme (ECM). This leads to excessive deposition of ECM and abnormal lung tissue repair [10]. This ultimately leads to scarring and fibrosis of the lung tissue [11], which in turn leads to respiratory failure and death [12]. Inflammation, fibrosis, and abnormal activation of immune cells play key roles in developing IPF [11]. Immunocytes, especially lung macrophages, play a regulatory role. Lung macrophages, one of the most abundant immune cells in healthy lungs [13], participate in various mechanisms contributing to IPF development and fibrosis processes, including maintaining lung homeostasis, clearing apoptotic cells, participating in wound healing, and initiating immune responses [14]. Macrophages exhibit strong plasticity and can differentiate into different subtypes, including classically activated macrophages (M1) and selectively activated macrophages (M2) [15]. Research indicates that M1 macrophages release pro-inflammatory cytokines and chemokines, such as tumor necrosis factor-alpha (TNF-α), interleukin-1 beta (IL-1β), and IL-12, inducing the occurrence of inflammatory reactions [16, 17]. In contrast, M2 macrophages do not produce pro-inflammatory cytokines and are associated with anti-inflammatory, repair, and fibrosis processes [18]. Activated M2 macrophages can secrete a range of fibrosis factors, such as transforming growth factor-beta (TGF-β), platelet-derived growth factor (PDGF), fibroblast growth factor (FGF), and IGF-1, promoting fibroblast proliferation and inducing differentiation into myofibroblasts [19]. Simultaneously, M2 macrophages release CCL18, stimulating fibroblasts to produce collagen. The feedback loop between fibroblasts and collagen further stimulates the continuous activation of M2 macrophages and excessive collagen production [20]. In addition to promoting inflammation and fibrosis processes, macrophages mediate inflammation and repair fibrosis, including clearing inflammatory mediators and cell debris, wound healing, and tissue repair [21]. Through single-cell level analysis, we can more comprehensively understand the complexity of IPF and individual differences. Therefore, in-depth research into the heterogeneity and function of macrophages in idiopathic pulmonary fibrosis will help reveal the disease's pathogenesis, providing a theoretical basis for developing more effective treatment strategies.

This study identified a significant increase in macrophages in IPF patients by analyzing a public single-cell RNA dataset (scRNA-seq). We then systematically classified macrophages to identify IPF-associated macrophage clusters and discovered a subtype of macrophages (ATP5-MΦ) present only in IPF lung tissue. Subsequently, through high-dimensional weighted gene co-expression network analysis (hdWGCNA), we identified co-expressed gene modules associated with IPF-MΦ. To further determine the value of these genes for disease prognosis, we incorporated them into machine learning to construct predictive models for assessing the prognosis of IPF patients. These findings not only provide new insights into the pathogenesis of IPF but also provide a substantial molecular and clinical basis for developing future therapeutic strategies.

## Materials and methods

### Data download

All study data were obtained from the Gene Expression Omnibus (GEO) database (http://www.ncbi.nlm.nih.gov/geo/). Specifically, idiopathic fibrosis scRNA-seq data were obtained from (GSE128033) [22], including 10 normal samples and 8 IPF samples (a total of 66,500 cells), a dataset with one of the largest number of samples we were able to retrieve at this time. To construct and validate our prediction model, we considered from a clinical point of view that disease samples have greater individual differences than normal samples. Therefore, this study prioritized using datasets with more disease samples rather than datasets with small differences in sample size between groups but small sample sizes. In the end, we chose data with larger sample sizes and more complete clinical information, including (dataset GSE32537:39NORMAL/131IPF) [23] to construct the model and (dataset GSE110147:11NORMAL/22IPF) [24] to be used as the validation set of the model.

### Initial processing of single-cell sequencing data

ScRNA-seq data was analyzed using the SeuratR package (V.4.3.0) [25]. Initially, quality control measures were implemented by filtering cells with mitochondrial gene expression > 10% and a gene expression level between 200 and 6000 genes. After quality control, SCTransform was applied to normalize and scale scRNA-seq data. Principal component analysis (PCA) was then performed on the processed data for dimensionality reduction. The Harmony method mitigated batch effects in the dissociated scRNA-seq data. The FindNeighbors function in Seurat generated a shared nearest-neighbor graph (NNG), and the FindClusters function used the Louvain algorithm for cluster analysis, visualized by tSNE scatter plots. To ensure the accuracy of cell annotation, the “FindMarkers” function was employed to identify genes preferentially expressed in each cluster and differentially expressed genes (DEG) between fibrotic and normal cells. Annotation of major cell clusters was performed using known cell type marker genes [22].

### Enrichment analysis and pseudotime trajectory

Based on DEGs, we conducted gene set enrichment analysis between cell subgroups using the clusterProfiler package [26] (version 4.7.1003), including single-sample gene set enrichment analysis (ssGSEA) and Gene Ontology (GO) enrichment analysis [27]. Finally, the “GseaVis” package (version 0.0.8) was used to visualize the functional enrichment results. To study the pseudotime trajectory of macrophages, we extracted the macrophage subgroups (a total of 16,872 cells) and used the DDR-Tree algorithm from the “Monocle” package (version 2.26.0) [28] to infer the developmental trajectory of macrophages.

### Cell–cell communication analysis and metabolism analysis

To comprehensively understand the interactions and communication between macrophages and other cell groups, the "CellChat" package (version 1.6.1) [29] was used to construct a cell–cell interaction network, following the recommended pipeline with default settings.

### High-dimensional WGCNA (hdWGCNA) analysis

hdWGCNA is a method for analyzing high-dimensional gene expression data, such as single-cell RNA-seq, to perform weighted gene co-expression network analysis (WGCNA) in high-dimensional data. We followed the standard procedure using the hdWGCNA package (version 0.1.1.9010) [25, 30]. Metacells were constructed separately for each sample and each cell cluster using the MetacellsByGroups function, with each metacell containing 50 cells. Macrophages were then extracted for a new Seurat, and subsequently, the TestSoftPowers, ConstructNetwork, ModuleEigengenes, ModuleConnectivity, and RunModuleUMAP functions were executed. The HubGeneNetworkPlot function was run for interaction analysis.

### Machine learning for predictive model construction

To evaluate the prognostic value of IPF-associated macrophage genes selected by hdWGCNA, three machine learning models were constructed and validated using LASSO analysis [31], random forest algorithm [32], and SVM-RFE algorithm [33]. The best lambda value for LASSO analysis was determined, and the top 49 genes were selected. The random forest algorithm assessed the importance of each feature, selecting the top 49 genes. Finally, the SVM-RFE algorithm was employed to extract the top 48 genes with importance. The intersection of the three machine learning-selected genes was used to build the predictive model. The “mlr3” package [34] (version 0.16.0) in R was used for machine learning model construction, including log_reg (logistic regression), LDA (linear discriminant analysis), ranger (random forest), SVM (support vector machine), naive_bayes (naive Bayes classifier), part (recursive partitioning and regression trees), and kknn (k-nearest neighbors). To determine the most accurate model, ROC curves were generated using the “timeROC” R package (version 0.4), and the predictive ability of each model was assessed. The final model’s predictive ability was also evaluated using an independent test set.

### IPF-MΦ module gene non-negative matrix factorization

The “NMF” package (version 0.26) [35] was used for non-negative matrix factorization of IPF. Feature genes identified by hdWGCNA for IPF-MΦ were selected from the GSE70866 dataset (20 normal, 212 IPF). Subsequently, the expression matrix of these genes was used for non-negative matrix factorization to identify different IPF subtypes.

### Immune microenvironment analysis

Immune landscape analysis was conducted using the GSVA package (version 1.44.5). The correlation between the 28 identified immune cell types and specific genes was visualized using the “corrupt” package (version 0.92) in the form of a heatmap. Additionally, the abundance of immune cell types was visualized using the “ggplot2” and “pheatmap” packages (version 3.4.1/1.0.12).

### Identification of IPF-MΦ feature genes affecting patient prognosis

To determine the impact of genes screened by machine learning on the prognosis of IPF patients, survival analysis was conducted using the “survival” package (3.2–13) and “survminer” package (0.4.9). Initially, univariate Cox regression analysis was performed to identify IPF-related macrophage genes (IRMG) influencing prognosis. Subsequently, based on the median gene expression, the samples in the GSE70866 dataset were divided into high and low-expression groups, and Kaplan–Meier (KM) survival analysis was conducted using the log-rank test to examine the differences in survival between these two groups. Furthermore, receiver operating characteristic (ROC) curve analysis was used to confirm the prognostic value of IRMG, with an area under the curve (AUC) greater than 0.7 indicating high diagnostic accuracy.

### Statistical analysis

All statistical analyses and data visualizations were performed using R software (version 4.2.1). For quantitative data, two-tailed, unpaired Student t-test or one-way analysis of variance (ANOVA) combined with Tukey’s multiple comparison test was used to compare means between two or more groups. Pearson’s correlation coefficient measured the linear correlation between two continuous variables. *p* < 0.05 was considered statistically significant.

## Results

### Single-cell sequencing analysis reveals cellular composition changes in IPF lung tissue

**Fig. 1:**
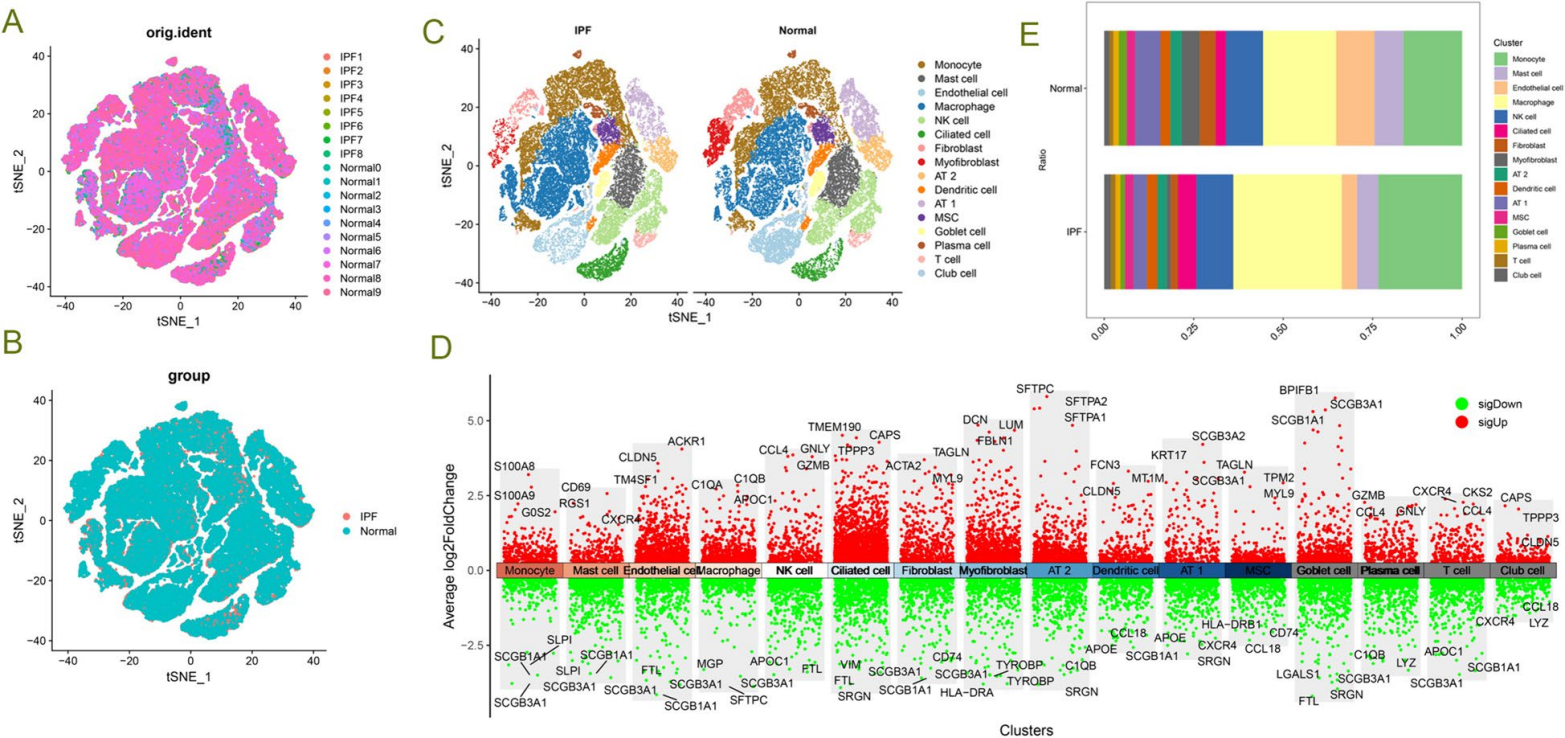
Changes in the Proportion of Macrophage Subtypes in Idiopathic Pulmonary Fibrosis (IPF) Lung. **A**, **B** t-SNE images showing cell distribution in normal and idiopathic fibrotic lung tissue. **C** Unbiased clustering divides cells into 16 cell clusters, and each cluster is distinguished by different coloring. **D** LogFC visualization of the top three genes for each cell type in normal and idiopathic fibrosis lung tissue after differential analysis. **E** Proportion of each cell type in normal and idiopathic fibrotic lung tissue

To explore the changes in cellular composition in IPF lung tissue, we collected and analyzed single-cell sequencing data from normal and IPF lung tissues. After quality control and batch effect correction, we obtained 66,500 qualified cells expressing 25,765 genes. Through unsupervised dimensionality reduction and clustering algorithms, we annotated and integrated cell types. Ultimately, we identified 16 cell clusters and visualized them using t-SNE (Fig. 1C), including monocytes (CD14 and CD16), mast cells (MS4A2 and CPA3), endothelial cells (VWF), macrophages (MACRO and MRC1), NK cells (KLRD1 and CD3E), ciliated cells (FOXJ1), fibroblasts (COL1A1), myofibroblasts (COL1A1, ACTA2, and PDGFRA), type II alveolar epithelial cells (SFTPB and SFTPC), dendritic cells (MHC II), AT-1 cells (AGER), mesenchymal stem cells (CD44 and ENG), goblet cells (MUC5B), plasma cells (CD79A), T cells (CD8 and CD4), and club cells (CYP2F2). Next, we investigated the changes in cellular composition in IPF lung tissue compared to normal. We found that monocytes and macrophages increased, while fibroblasts, myofibroblasts, and AT1 were significantly reduced (Fig. 1E, Supplementary Fig. 1). Furthermore, we analyzed differentially expressed genes for each cell type (Fig. 1D), with the top 3 upregulated and downregulated genes in macrophages being C1QA/C1QB/AP0C1 and MGP/SCGB3A1/SFPTC, respectively.

### Single-cell analysis reveals macrophage heterogeneity in IPF lung tissues

**Fig. 2:**
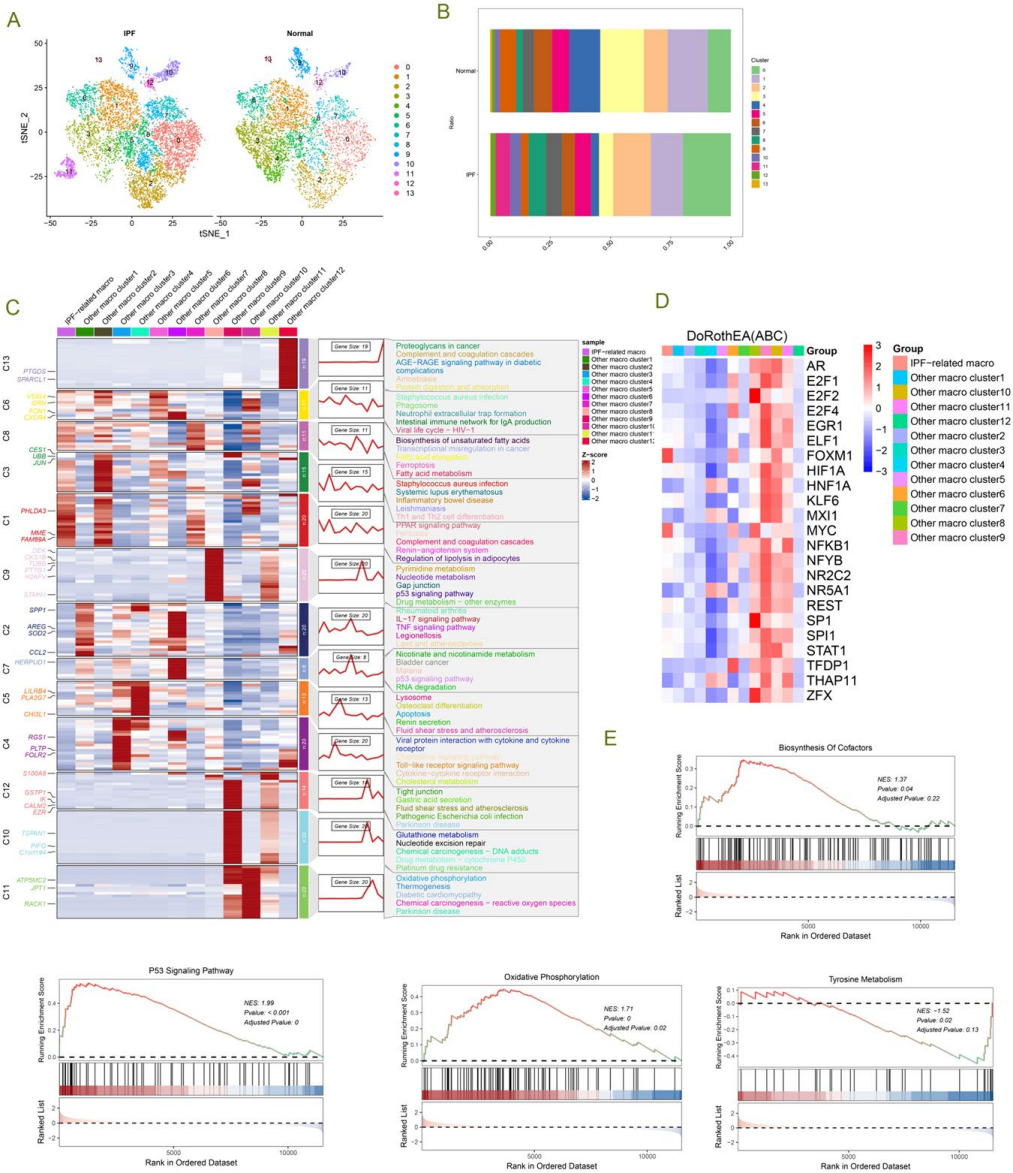
Single-cell analysis reveals the heterogeneity of macrophages in idiopathic pulmonary fibrosis (IPF). **A** t-SNE plot displaying the distribution of 13 subtypes of macrophages in normal and IPF lung tissues. **B** Proportions of macrophage subtypes in IPF compared to normal tissues. **C** Left panel: Dynamic patterns of representative differentially expressed genes (DEGs) in each macrophage cluster are illustrated in this series of graphs. Middle panel: Heatmap depicting the representative DEGs between each cell cluster. Right panel: Representative enriched gene ontology (GO) terms for each cluster. **D** Heatmap displaying the gene changes in each macrophage subtype. **E** Representative gene set enrichment analysis (GSEA) pathways in IPF-associated macrophages, including pathways related to cofactor biosynthesis, p53 signaling, oxidative phosphorylation, and tyrosine metabolism

To further explore specific changes in macrophages, we extracted macrophages and further classified them into 13 subclusters based on macrophage-specific highly variable genes (Fig. 2A, B). We observed that Clusters 0, 2, 7, 8, 10, and 12 were increased in IPF lung tissues compared to normal lung tissues, with Cluster 0 identified as IPF-related macrophages (IPF-MΦ). In contrast, Clusters 1, 3, 4, 5, 9, and 13 were decreased, and interestingly, Cluster 11 was exclusively present in IPF lung tissues. We conducted a separate analysis of Cluster 11 (Supplementary Fig. 2). In this cluster, we found high expression of the ATP5 gene family, including ATP5F1E, ATP5MG, ATP5MC3, ATP5MC2, SMIM25, ATP5MPL, ATP5MD, ATP5MF, ATP5F1D, etc. (Supplementary Table 1). We confirmed through the HUGO Gene Nomenclature Committee (HGNC, https://www.genenames.org/) database that these genes belong to the ATP synthase subunit genome [36]. Based on the expression profile of this genome, Cluster 11 was named ATP5- MΦ.We analyzed the differentially expressed genes in ATP5-MΦ and generated a volcano plot (Supplementary Fig. 2A). Next, we performed enrichment analysis on these differentially expressed genes. GO enrichment analysis revealed (refer to Supplementary Fig. 2B) that these differentially expressed genes were mainly involved in biological processes such as proton transmembrane transport, proton-transporting two-sector ATPase complex, and proton transmembrane transporter activity. GSEA analysis showed enrichment of upregulated oxidative phosphorylation and energy production gene sets in the entire gene set (Supplementary Fig. 2C), while the IL-17 signaling pathway gene set was downregulated.

To explore the gene expression data of each macrophage subtype at the single-cell level, we calculated the relative expression levels of each gene in each individual cell and assigned a score to each gene. Subsequently, we performed unsupervised clustering of these genes, forming different gene clusters (Fig. 2C). We grouped genes with similar expression trends, obtaining a consensus of 13 different expression trends related to various biological functions, as shown in the clustering results in Fig. 2C. We observed that genes in Cluster 1 of IPF-MΦ exhibited significantly high expression levels, and these genes were highly enriched in biological functions such as Th1 and Th2 cell differentiation, PPAR signaling pathway, pertussis, complement, and coagulation cascades, renin-angiotensin system, and regulation of fat breakdown in adipocytes, among others. The differential gene heatmap of the 13 macrophage subclusters is displayed in Fig. 2D, where FOXM1 and MYC were highly expressed in IPF-MΦ, and the expression levels of Cluster 8, 9, 10, and 11 differential genes were similar. Through GSEA analysis (Fig. 2E), we found an upregulated enrichment of accessory factor synthesis and p53 signaling pathway gene sets in IPF-MΦ, while oxidative phosphorylation and tyrosine metabolism gene sets showed a downregulated enrichment.

### Pseudo-time trajectory analysis and cell communication analysis of IPF-associated macrophages

**Fig. 3:**
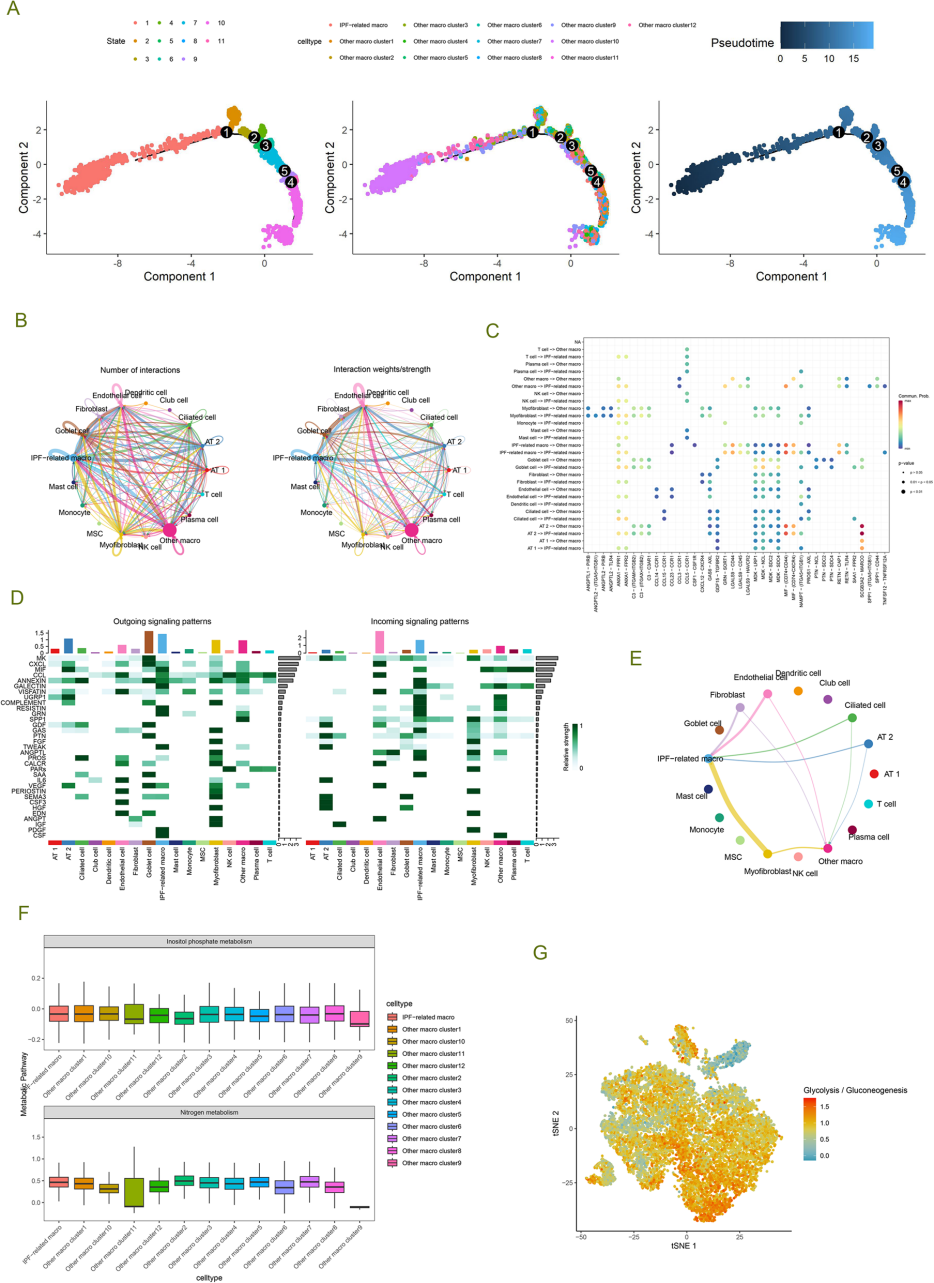
Pseudo-temporal trajectory and intercellular communication analysis of IPF-MΦ. **A** Pseudo-temporal trajectory analysis illustrates branching points and directions of macrophage differentiation in idiopathic pulmonary fibrosis (IPF). **B** CellChat analysis-based visualization of communication patterns among all cell types, including the number and strength of interactions. **C** Probability of ligand-receptor-mediated communication between macrophage subtypes and other cell populations. **D** Heatmap displaying differences in the strength of incoming and outgoing interactions between each cell type. **E** Signaling network based on differential analysis, with arrow direction indicating the direction of cell communication. **F** Bar chart quantifying Inositol phosphate metabolism and Nitrogen metabolism in macrophages. **G** t-SNE visualization depicting the levels of glycolysis and gluconeogenesis in macrophages

We delved into the developmental trajectory of macrophages and the communication network between macrophages and other cell types in IPF through pseudo-time trajectory analysis and CellChat analysis. Pseudo-time trajectory analysis revealed the unique position of IPF macrophages on the developmental trajectory (Fig. 3A). The formation of IPF macrophage cluster 10 was observed in the initial stages of the trajectory. IPF-MΦ was discovered at trajectory branches 3, 4, and 5, suggesting a specific developmental process for IPF macrophages. Subsequently, CellChat analysis was employed to understand further the intricate communication network between IPF-MΦ and surrounding cell clusters. The results indicated that IPF-MΦ exhibited outstanding communication capabilities with dense interactions with various cell types (Fig. 3B). Particularly noteworthy was the intense interaction of IPF-MΦ with other macrophages through various ligand receptors, such as GRN-SORT1 and MIF-(CD74 + CD44) (Fig. 3C). The strength of both incoming and outgoing interactions of IPF-MΦ was higher compared to other macrophages (Fig. 3D). Signal pathways like MIF, ANNEXIN, GALECTIN, and VISFATIN showed similar patterns in both incoming and outgoing signals, representing the utilization of similar signaling transduction pathways for incoming or outgoing signals. The pathways with the highest incoming and outgoing signal strengths for IPF-MΦ were MK and cxcl signaling pathways.

Further differential analysis revealed that, compared to other macrophages, IPF-MΦ exhibited significantly enhanced communication with myofibroblasts, ciliated cells, AT2, epithelial cells, and fibroblasts (Fig. 3E). This enhanced communication might be closely related to the emergence of the ANXA1-FPR2 and NAMPT-(ITGA5 + ITGB1) signaling pathways in IPF-associated macrophages (Fig. 3C). Activating these signaling pathways might play a crucial role in the pathogenesis of IPF, influencing interactions and communication between different cell types thereby affecting inflammation, immune responses, and potential fibrotic processes. Additionally, we analyzed the metabolic levels of macrophage subtypes (Fig. 3F, G). Compared to other macrophage subtypes, Cluster 9 and 11 showed lower levels of nitrogen and inositol phosphate metabolism, while Cluster 10 exhibited lower levels of glycolysis and gluconeogenesis. These differences in metabolic levels may be related to the functional distinctions among macrophage subtypes in IPF.

### hdWGCNA reveals module hub genes in IPF-MΦ

**Fig. 4:**
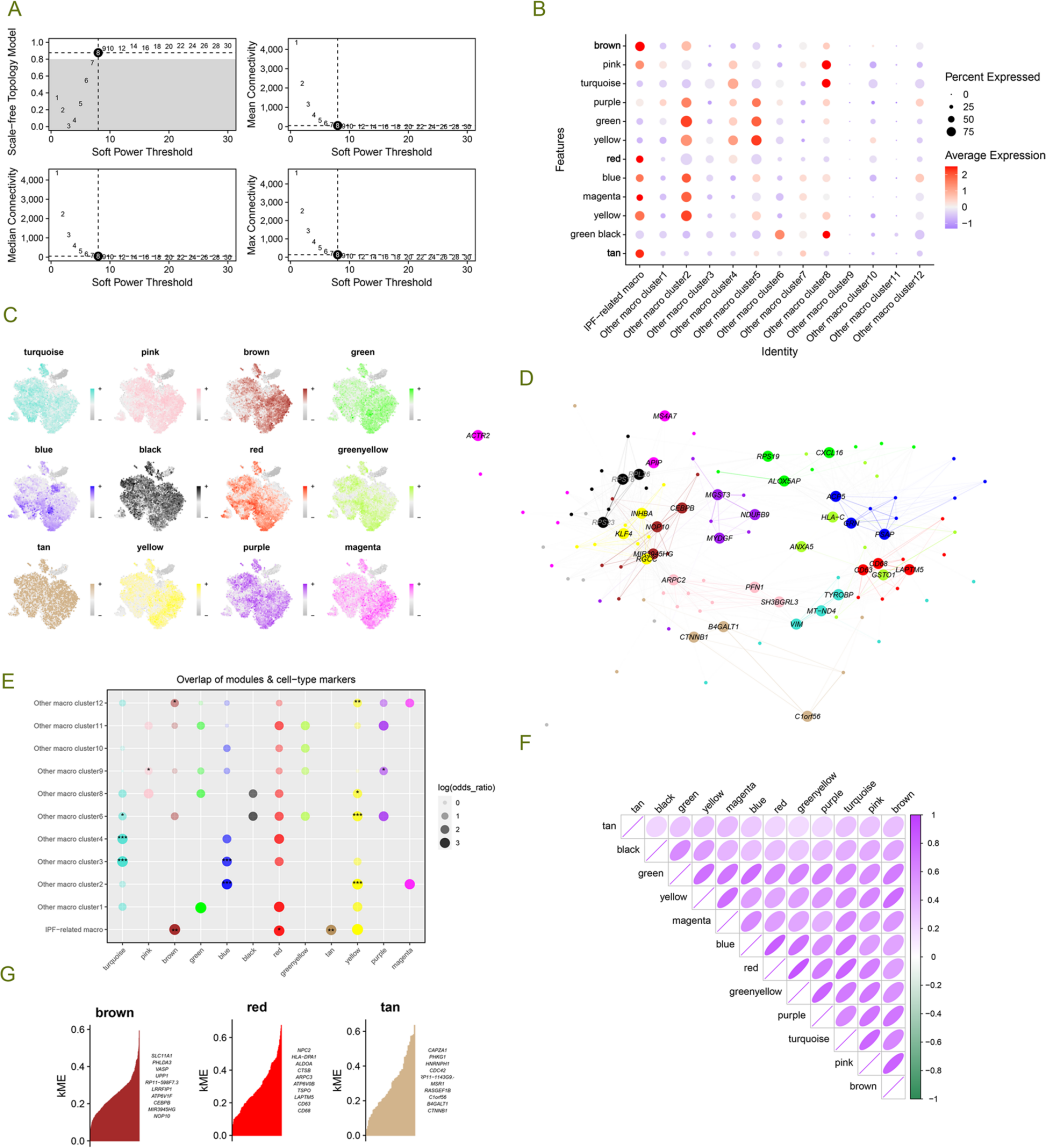
hdWGCNA Identifies Module Hub Genes for IPF-MΦ. **A** The average connectivity plot displays the scale-free topology fitting index and soft-thresholding ability (optimal soft-threshold value is 8). **B** Estimation of module activity in different macrophage clusters using the hdWGCNA algorithm. **C** t-SNE plot illustrating the expression distribution of each module hub gene across 13 macrophage clusters. **D** For each module detected by WGCNA, a protein–protein interaction (PPI) network was constructed by extracting the top 10 genes from each module. **E** Gene overlaps within different modules. **F** Presentation of the module-to-module relationship matrix based on the correlation of module hub genes. **G** Brown, red, and tan modules are gene modules for IPF-MΦ, and the top 10 hub genes are presented according to the hdWGCNA process. * *p* < 0.05, ** *p* < 0.01, *** *p* < 0.001

To unravel the potential functions of the IPF macrophage subtype, we employed high-dimensional weighted gene co-expression network analysis (hdWGCNA). We chose a power of 8 to construct a scale-free network, resulting in 12 gene modules (Supplementary Fig. 3A, Fig. 4A-C). Interestingly, the purple, green, and yellow modules exhibited substantial expression in cluster 2 and cluster 5 macrophages, while the blue, magenta, and yellow-green modules were highly expressed in IPF-MΦ and cluster 2 macrophages. We visualized each module's protein–protein interaction (PPI) networks based on the top 10 hub genes within the 12 gene modules (Supplementary Fig. 3B, Fig. 4D).

**Fig. 5:**
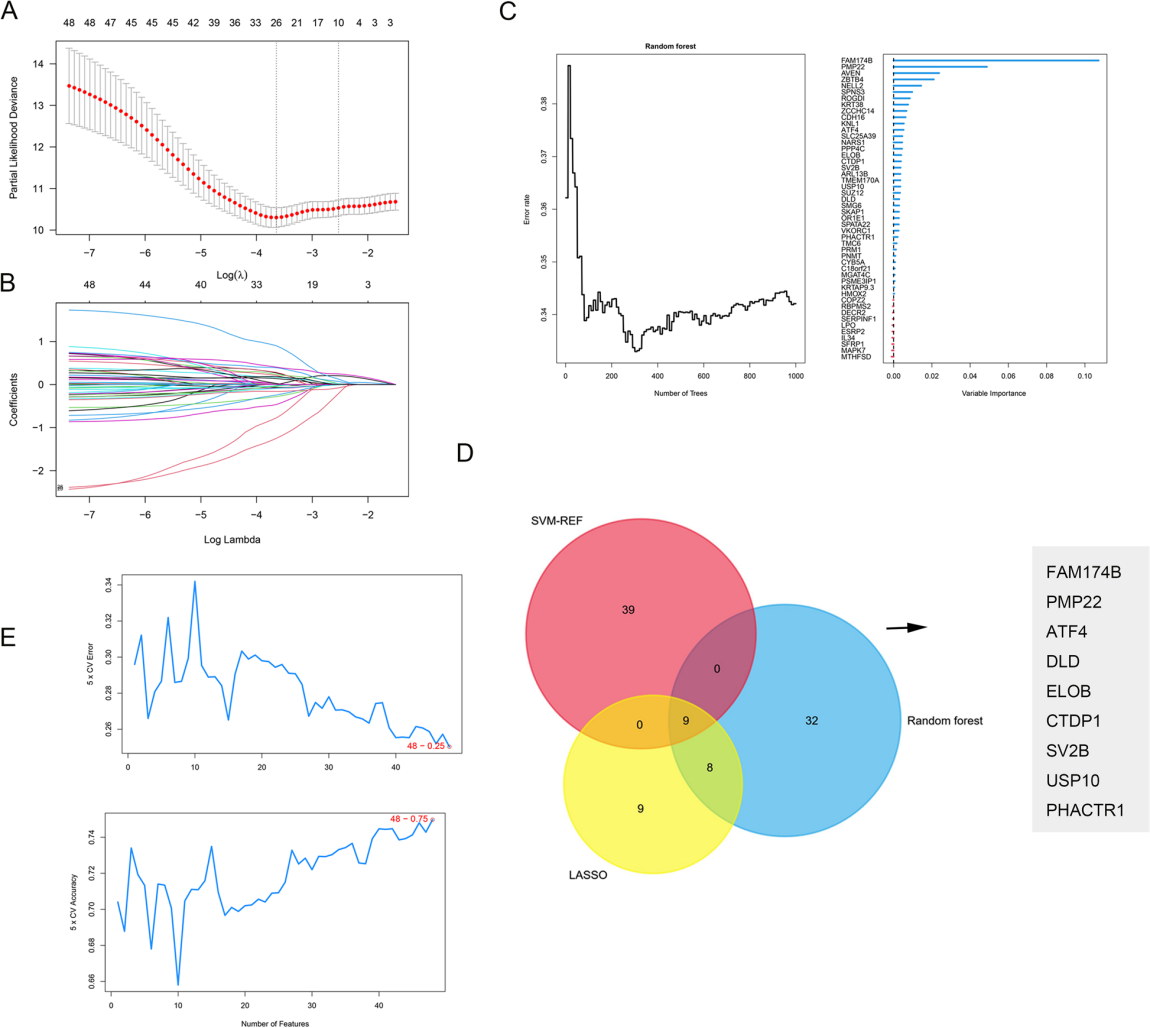
Various machine learning methods identify key genes associated with macrophages and fibrosis progression. **A**,**B** LASSO regression was used to screen and identify 26 key genes. **C** Random forest ranks all genes to determine their importance in the model. **D** Venn diagram filtering reveals overlapping genes from the three algorithms, including FAM174B, PMP22, ATF4, DLD, ELOB, CTDP1, SV2B, USP10, and PHACTR1. **E** The support vector machine recursive feature elimination (SVM-RFE) method was used to evaluate all genes and the genes were ranked based on the average ranking

Further exploration of the inter-module relationships (Fig. 4F) revealed a robust positive correlation between the red module and the blue and green-yellow modules. Moreover, the brown, red, and tan modules exhibited a preference for expression in IPF-MΦ and displayed significant correlations (Figs. 4B and 5E). We identified the genes within the brown, red, and tan modules as characteristic genes of IPF-MΦ and constructed PPI networks using the hub genes from each module (Supplementary Fig. 3C).

### Various machine learning algorithms reveal signature genes for IPF-MΦ

We constructed predictive models using the hub genes from the brown, red, and tan modules to further explore the relationship between hub genes of IPF-MΦ gene modules and the onset and progression of IPF. Employing various machine learning methods to reduce the false-positive rate of screening results, we conducted a detailed analysis of the RNA dataset (GSE32537). Initially, through the LASSO regression algorithm, we successfully identified 26 key genes closely associated with the prognosis of IPF patients (Fig. 5A, B). Subsequently, these genes were ranked using random forest analysis (Fig. 5C), and the top 30 genes in importance were extracted. Using SVM-RFE methodology, we evaluated all genes through tenfold cross-validation and obtained the top 30 important genes based on their average rankings (Fig. 5E). Finally, we generated a Venn diagram to identify overlapping genes from the three machine learning methods (Fig. 5D), resulting in nine significant genes, including FAM174B, PMP22, ATF4, DLD, ELOB, CTDP1, SV2B, USP10, and PHACTR1, all closely associated with the prognosis of IPF patients.

### Relationship between IRMG and the immune microenvironment

**Fig. 6:**
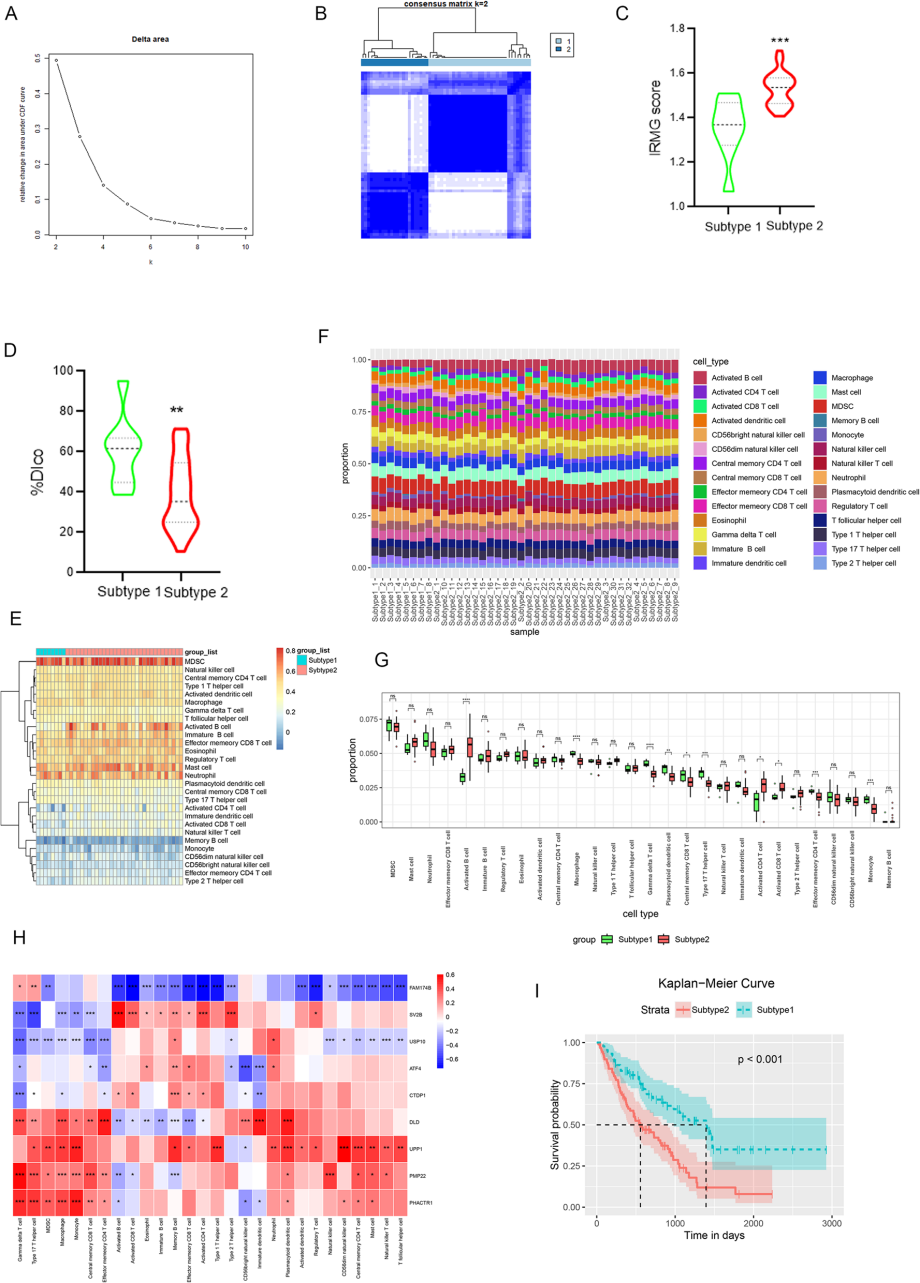
Immunoinfiltration analysis. **A** Cumulative Distribution Function (CDF) curve assesses the metric indicating differences or improvements in performance between two models or methods. **B** The unsupervised NMF algorithm divides IPF patients into two groups. **C** Violin plots display the differences in IPF-MΦ characteristic gene (IFMG) scores between the two groups, as well as (**D**) the Diffusion Capacity of the Lungs for Carbon Monoxide (%DLCO). **F** Stacked bar charts depict the composition of immune cells in different subtypes. **E** Heatmap and (**G**) bar charts show the relative proportions of different immune cell types in high-risk and low-risk patients. **H** Heatmap displays the correlation between IFMG and immune cells. **I** Kaplan–Meier curves for high-risk and low-risk patients. * *p* < 0.05, ** *p* < 0.01, *** *p* < 0.001

To elucidate the relationship between the gene characteristics of IPF-MΦ and the immune microenvironment, we employed CDF curve analysis to determine the effectiveness of this algorithm in patient grouping (Fig. 6A). The apparent inflection points on the curve at different values suggested optimal algorithm performance when patients were divided into two groups. Subsequently, for a more comprehensive understanding of the biological characteristics of these two subtypes and their roles in the development of IPF, we utilized the differentially expressed IPF-MΦ characteristic genes (IRMG) and the NMF algorithm to cluster IPF patients into two subtypes (see Fig. 6B). We then assessed IRMG scores and the Diffusion Capacity of the Lungs for Carbon Monoxide (DLCO) index for the two subtypes (see Fig. 6C, D). It was found that patients with type 2 had significantly higher IRMG scores and significantly lower DLCO indices compared to patients with type 1, which is a measure of lung function, so patients with type 2 had poorer lung function. Additionally, we analyzed the immune microenvironment of the two subtypes (see Fig. 6E-G). In comparison to Type 1 IPF patients, Type 2 IPF patients showed a significant increase in the proportions of macrophages, γδ T cells, plasmacytoid dendritic cells, central memory CD8 T cells, type 17 T cells, effector memory CD4 T cells, and monocytes. Conversely, the proportions of activated B cells, activated CD4 T cells, and activated CD8 T cells were significantly elevated. These findings suggest distinct immune response patterns between Type 1 and Type 2 IPF patients, with Type 2 IPF patients potentially exhibiting a more activated and inflammatory immune state.

Furthermore, these nine genes exhibited a strong correlation with immune cells. Notably, different genes showed varying correlations with immune cells, and even opposite results were observed. For example, FAM174B exhibited a significant negative correlation with most immune cells, while UPP1 showed a significant positive correlation with most immune cells. We posit that this may be related to these genes’ different roles in various immune regulatory pathways.

### Machine learning to build a predictive model

**Fig. 7:**
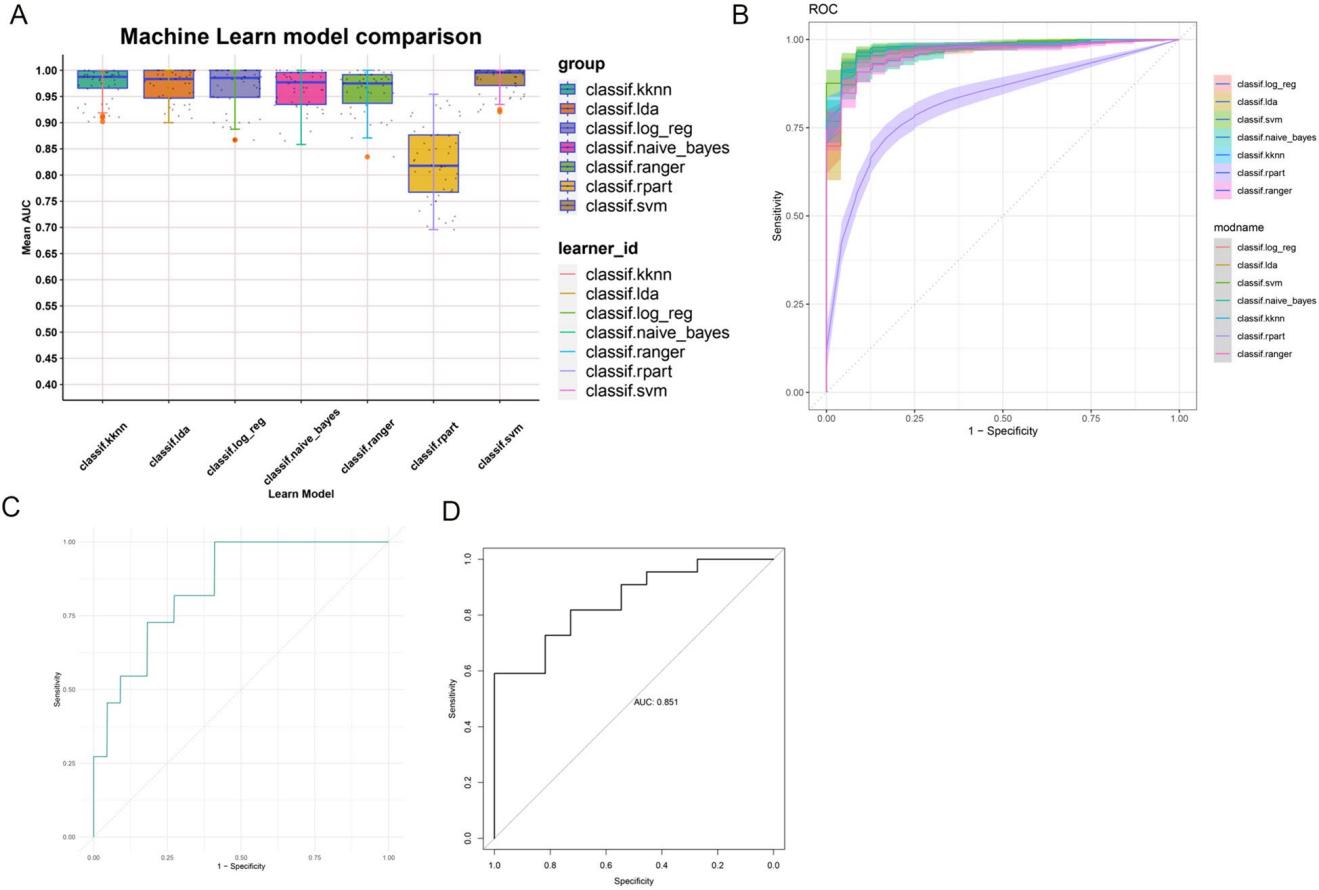
Machine learning to build a predictive model. **A** Construction of predictive models using seven machine learning algorithms. **B** Average AUC values from 10 repetitions of 5-fold cross-validation for each model. **C** ROC curves of the model in the independent external validation set GSE110147. **D** ROC curves of the model in the independent external validation set GSE70866

Based on the identified IFMG through machine learning, we constructed a predictive model for predicting the onset and progression of IPF in patients. We employed seven machine learning algorithms from the mlr3 package to find the most optimal algorithm. When evaluating the performance of these models, using the Area Under the Curve (AUC) as the metric, the Support Vector Machine (SVM) machine learning algorithm exhibited the highest accuracy, sensitivity, and specificity (Fig. 7A, B). Subsequently, we validated the performance of the predictive model using the GSE110147 and GSE70866 datasets, yielding AUC values of 0.893 (Fig. 7C) and 0.851 (Fig. 7D), indicating that the model can effectively distinguish between high-risk and low-risk IPF patients. This series of work has yielded more effective clinical diagnostic markers and provided important clues for future diagnostic and therapeutic research.

### FAM174B, PHACTR1, DLD and ATF4 identified as genes influencing the prognosis of IPF patients

**Fig. 8:**
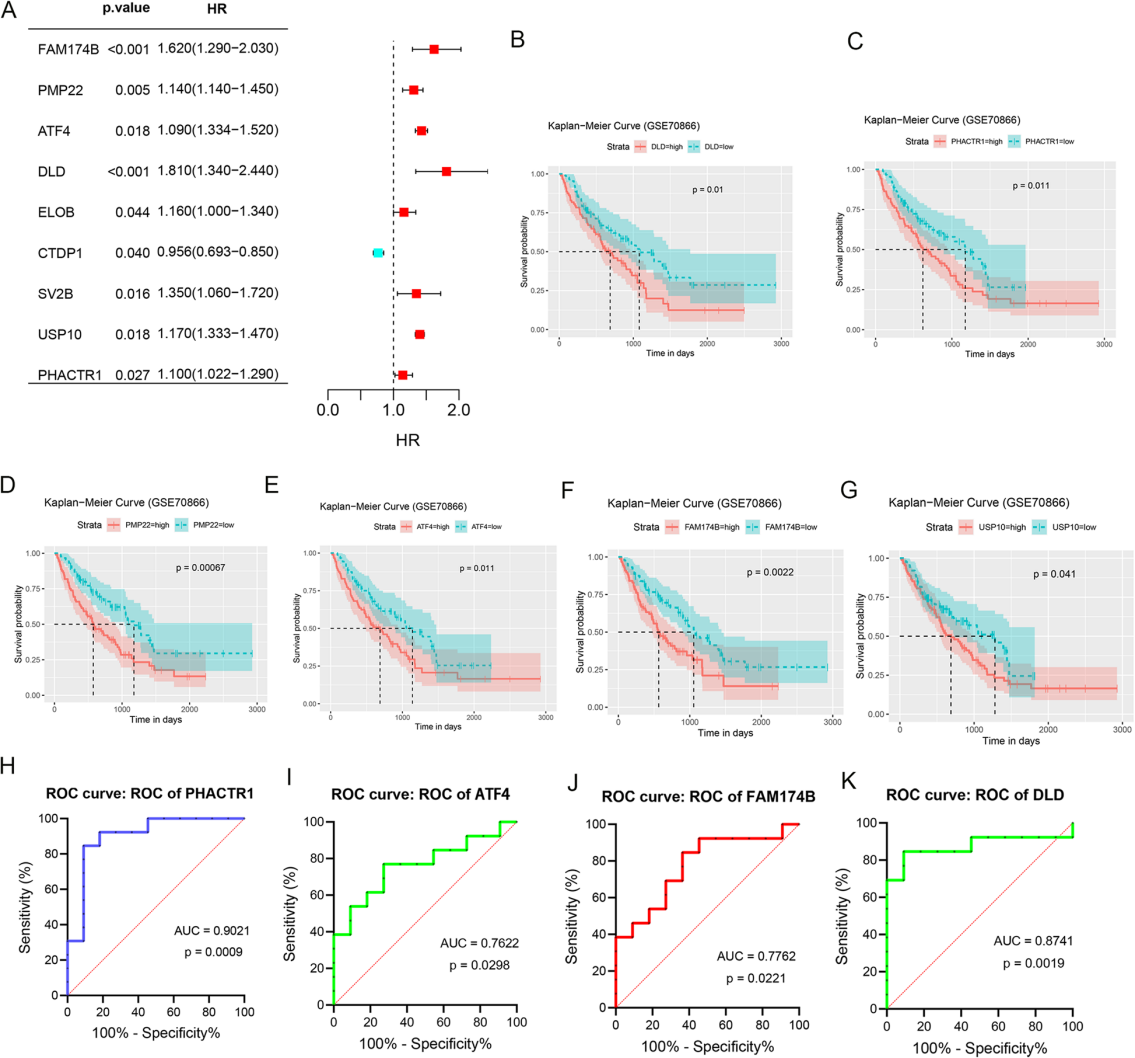
Further validation of genes influencing the prognosis of IPF patients. **A** Screening genes for inclusion in prognostic analysis by univariate COX regression. **B**-**G** Kaplan–Meier survival analysis was used to evaluate the relationship between the expression of DLD, PHACTR1, PMP22, ATF4, FAM174B, and USP10 genes and patient survival time. ROC curves for (**H**) PHACTR1, (**I**) ATF4, (**J**) FAM174B, and (**K**) DLD external data set validation

To confirm the association of these nine genes with the prognosis of IPF patients, we subjected these significant genes to univariate Cox regression analysis and Kaplan–Meier (KM) survival analysis. The Cox regression analysis results (Fig. 8A) indicated that FAM174B, PMP22, ATF4, DLD, ELOB, SV2B, USP10, and PHACTR1 were associated with a higher risk of prognosis, while CTDP1 was linked to a lower risk. In KM survival analysis, we observed that DLD, PHACTR1, PMP22, ATF4, FAM174B, and USP10 were correlated with patient prognosis (Fig. 8B-G). Finally, ROC curves illustrated (Fig. 8H-K) that PHACTR1 (AUC = 0.9021, *P* = 0.0009), ATF4 (AUC = 0.7622, *P* = 0.0298), FAM174B (AUC = 0.7762, *P* = 0.0221) and DLD (AUC = 0.8741, *P* = 0.0019),might serve as valuable biomarkers.

## Discussion

The pathogenesis of IPF has not been fully elucidated but has been associated with various factors, including genes, environmental exposures, and chronic lung injury [1]. In this complex disease context, macrophages, as an important component of the immune system, play a crucial regulatory role in the pathology of IPF [14]. In the present study, we found significant changes in the cellular composition of lung tissues from both normal and IPF patients, particularly a significant increase in the percentage of macrophages. A specific class of macrophage subtype (ATP5-MΦ) was further identified, which was only present in IPF lung tissues. Subsequently, by hdWGCNA, we identified the co-expressed gene modules with IPF-MΦ and used machine learning to construct a model that predicts the survival prognosis of IPF patients.

Previous studies, in a mouse model of fibrosis [37] and patients with IPF [38], have observed that monocytes can be recruited to lung tissue to differentiate into macrophages, increasing infiltrating macrophages. The present study similarly found a significant increase in the number of monocytes and macrophages in IPF, which reflects an abnormal activation of the immune system and an exacerbation of the inflammatory process [19, 39]. In contrast, there was a significant decrease in the number of fibroblasts, myofibroblasts, and AT1 cells, which represents the destruction of the structure of the lung tissue and an acceleration of the fibrotic process [9, 40–42]. In the present study, a significant increase in monocytes and macrophages in IPF was observed in a mouse model of IPF and patients with fibrosis. IPF lung tissue shifted from homeostatic equilibrium to IPF fibroblasts, myofibroblasts, and abnormal basal-like cells [43]. Macrophages play an important regulatory role in IPF development, and we found that up-regulated genes in macrophages, including C1QA, C1QB [44], and APOC1 [45], may be associated with immune regulation and inflammatory response, while down-regulated genes, including MGP [46], SCGB3A1 [47], and SFPT [48] may be involved in abnormalities of fibrosis and tissue repair. Then, this study continued with an in-depth analysis of macrophages and found that IPF-related macrophage differential genes were mainly enriched in biological functions such as Th1 and Th2 cell differentiation and PPAR signaling pathways, which are closely related to the development of pulmonary fibrosis. Th1 cells and their secretions are thought to have antifibrotic effects, whereas Th2 cells can lead to lung tissue injury and promote fibrotic effects [46, 49]. The PPAR signaling pathway can intervene in TGF-β-induced fibroblast differentiation, and up-regulation of the signaling pathway can be anti-fibrotic. Macrophage subpopulations not present in normal lungs may promote fibrosis. Reclustering identified Cluster 11 as a macrophage subtype (ATP5-MΦ) specifically present in IPF, characterized by high expression levels of related genes encoding subunits of the mitochondrial ATP synthase complex. Through functional enrichment and GSEA of the differential genes of ATP5-MΦ, we reveal that ATP5-MΦ is active in proton transmembrane transport, ATPase complex, and oxidative phosphorylation. The interconnection between proton transmembrane transport and oxidative phosphorylation processes is particularly interesting. Proton transmembrane transport is a process that regulates the difference in proton concentration between inside and outside the cell, whereas oxidative phosphorylation is an important metabolic pathway for generating cellular energy [50]. ROS generated during oxidative phosphorylation promotes the progression of IPF. Anti-inflammatory macrophages usually exhibit lower metabolic activity and are more inclined to undergo oxidative phosphorylation to generate energy [51, 52]. This macrophage metabolic reprogramming may be related to their specific function in regulating immune responses.

The present study further investigated the interaction of IPF-MΦ with other cells in lung tissues. The interaction of ligand receptors such as GRN-SORT1 and MIF-(CD74 + CD44) may play an important role in the communication between IPF-MΦ and other macrophages. It has been reported that macrophage migration inhibitory factor (MIF) inhibits random migration and adhesion of monocytes/macrophages and regulates immune responses through activation of signaling pathways such as AKT and NF-KB by CD74/CD44 [53, 54]. GRN-SORT1, which is closely related to signaling for lipid metabolism, promotes macrophage cholesterol accumulation [55]. In addition, the role of IPF-MΦ in communicating with myofibroblasts, fibroblasts, ciliated cells, ciliated cells, and macrophages may be important in communicating with IPF-MΦ and other macrophages. Fibroblasts, ciliated cells, AT2, epithelial cells, and fibroblasts, and the enhanced communication may be closely related to the activation of specific signaling pathways such as ANXA1-FPR2 and NAMPT-(ITGA5 + ITGB1). Previous studies have found that the ANXA1-FPR2 signaling axis maintains fibroblast homeostasis [56]. ANXA1, which is also overexpressed in macrophages infiltrating damaged tissues, promotes an anti-inflammatory macrophage phenotype and inhibits inflammation and myofibrillar regeneration by activating the FPR2-AMPK signaling axis [57]. These results suggest that IPF-MΦ may regulate other immune cells in the microenvironment by promoting fibrosis.

We constructed and analyzed co-expression networks in scRNA-seq by hdWGCNA to obtain co-expressed gene modules of IPF-MΦ. Applying machine learning algorithms provides a powerful tool for screening and validating key genes in large-scale datasets. We used multiple machine learning algorithms to improve the reliability and generalization ability of the model and finally screened nine significant genes (IRMG), including FAM174B, PMP22, ATF4, DLD, ELOB, CTDP1, SV2B, USP10, and PHACTR1. Notably, patients with high IRMG scores had significantly decreased DLCO index, which suggests that patients may be accompanied by more severe lung function impairment. Moreover, patients with high IRMG scores present a more activated and inflammatory-prone immune state, which can affect disease progression and response to therapy [19, 58]. This study also found a strong correlation between IRMG and immune cells, and different genes showed diverse correlations with immune cells. For example, FAM174B was significantly negatively correlated with most immune cells, whereas UPP1 positively correlated with most immune cells. These results further emphasize that IRMG plays an important role in pulmonary fibrosis. Then, we constructed machine learning models based on 9 central genes for predicting the survival prognosis of IPF patients. After several validations, we selected the SVM model with the best accuracy, sensitivity, and specificity. Validated by a validation cohort, we confirmed that the prediction model constructed based on the construction of IPF-MΦ-related genes has good accuracy. This means that our prediction model can effectively predict the prognosis of IPF patients. Finally, this study further identified IRMGs that affect the prognosis of IPF patients. Ultimately, four core genes, FAM174B, PHACTR1, DLD, and ATF4, were identified as genes that affect the prognosis of IPF patients. Previous studies have reported the role of these genes in other diseases. For example, PHACTR1 can be involved in the regulation of atherosclerosis by affecting macrophage agonism and cytophagy [59, 60]. ATF4 promotes amino acid synthesis and protein degradation to maintain cellular homeostasis when cells are exposed to oxidative stress or nutrient deficiencies [61]. However, more experimental validation and in-depth studies are needed to confirm their exact functions in IPF.

This study also has some limitations. First, in vitro experiments are needed to further screen and identify biomarkers before identifying prognosis-related genes as feasible biomarkers. In the future, we will carry out relevant experiments to validate the above results and further explore the regulatory mechanisms and clinical significance of the new subgroups of macrophages, as well as FAM174B, PHACTR1, DLD, and ATF4, in the progression of the disease. Second, the pathogenesis of IPF is very complex, and the present study only focused on the analysis of macrophages without an in-depth study of other cell types. Further, the limited sample size included in this study is a limitation. Although we ensured the robustness of our findings through multiple independent datasets, there was some imbalance in the samples between the selected dataset groups. In addition, although we performed the ScRNA-seq analysis according to best practices [62], data quality and batch effects could potentially impact the results.

## Conclusion

In conclusion, this study has made a series of useful findings based on an in-depth study of macrophage subtypes and related gene expression patterns in IPF. We identified IPF-associated macrophage subpopulations by recluster analysis of macrophages. Then, we screened the genes affecting the prognosis of IPF patients based on machine learning and constructed a prediction model. The prediction model has high accuracy, sensitivity, and specificity. In addition, we identified a new macrophage subtype, ATP5-MΦ, and elucidated its possible important role in promoting fibrosis. This study provides new insights into the pathogenesis of IPF and personalized clinical treatment.

### Supplementary Information

Supplementary Material 1. 

Supplementary Material 2. 

## Data Availability

No datasets were generated or analysed during the current study.
