# Supplementary material for: IPF-related new macrophage subpopulations and diagnostic biomarker identification - combine machine learning with single-cell analysis
Source: Respir Res. 2024 Jun 13;25:241. doi: 10.1186/s12931-024-02845-8 (PMC11170785; doi:10.1186/s12931-024-02845-8)
Supplement: Supplementary file 1 — Supplementary Material 1. [file 12931_2024_2845_MOESM1_ESM.docx]

Supplementary Fig. 1 Box plots of percentage cell occupancy in IPF and normal lung tissue.


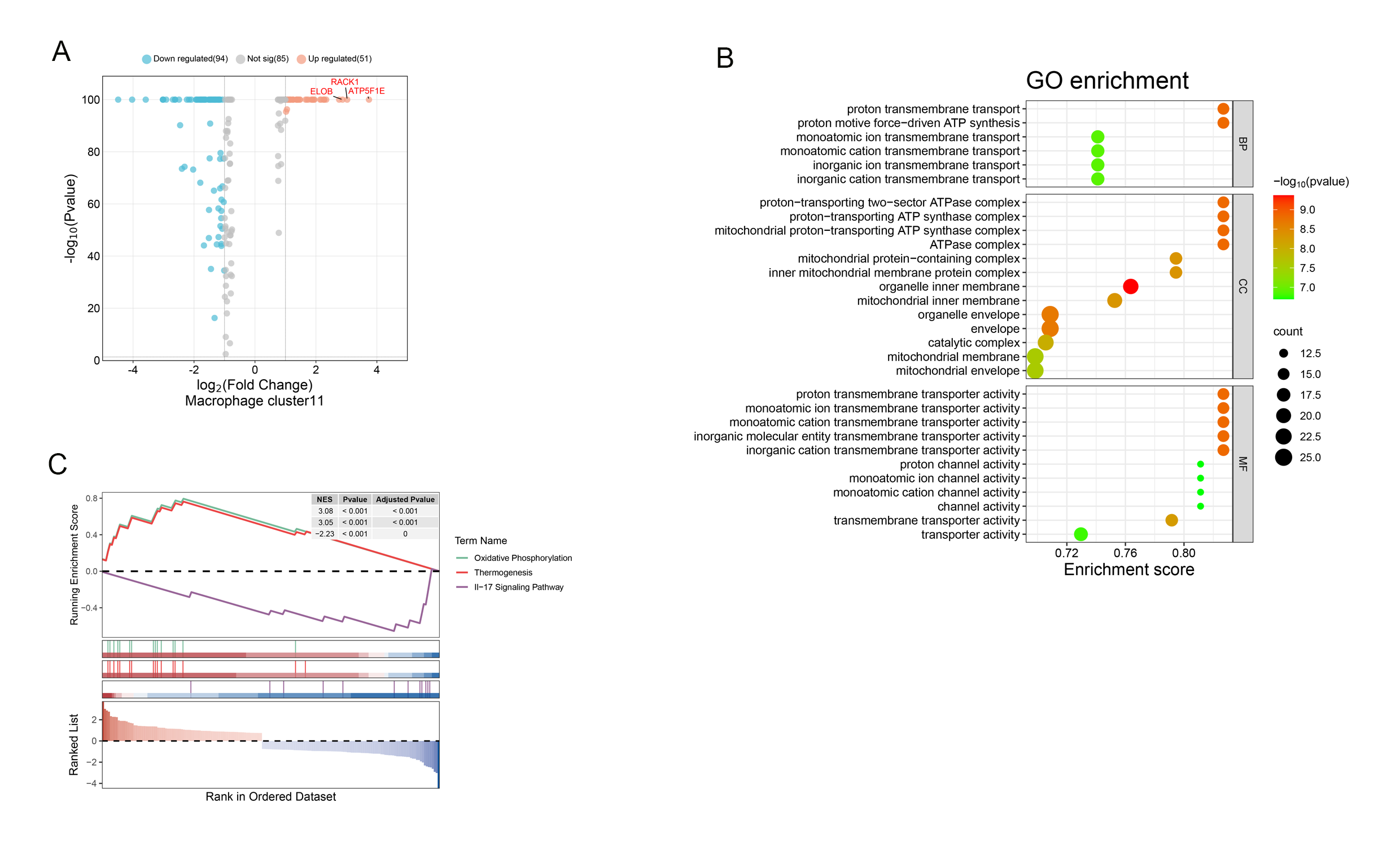


Supplementary Fig. 2 (A) Volcano plot of differentially expressed genes in ATP5-MΦ. (B) Gene Ontology (GO) enrichment analysis of upregulated genes. (C) Gene Set Enrichment Analysis (GSEA) of differentially expressed genes in ATP5-MΦ.


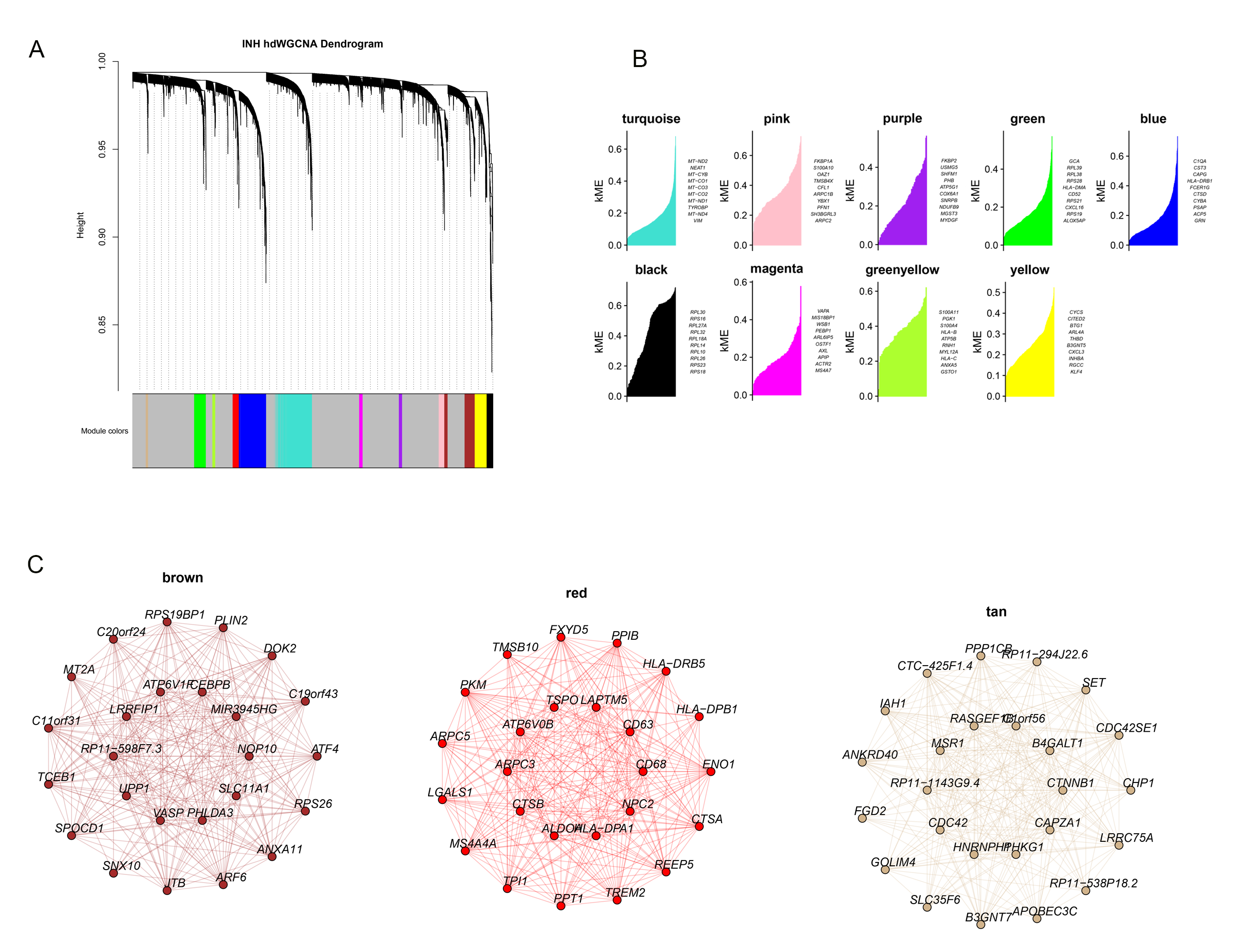


Supplementary Fig. 3 (A) The dendrogram based on Weighted Gene Co-expression Network Analysis (WGCNA) illustrates the expression patterns of genes, achieving hierarchical clustering of genes. Each leaf represents an individual gene, and the colors specified at the bottom indicate their membership in specific co-expression modules. Genes not grouped into any co-expression module constitute the "gray" module. (B) The kME plot displays the top 10 hub genes in each module ranked by their kME values in macrophages. Higher connectivity or kME values indicate that genes are more central or influential in their respective modules. (C) Protein-protein interaction (PPI) networks of hub genes in the brown, red, and tan color modules.
